# The Causes and Prevention of Yellow Fever

**Published:** 1856-01

**Authors:** 


*The Causes and Prevention of Yellow Fever, contained in the report of the Sanitary Committee of New Orleans.* By E. H. Barton, A. M., M. D., &c. Philadelphia—Lindsay & Blakison, 1855.

We are also indebted to the enterprising publishers for this work, which we shall notice after examining thoroughly. It seems to show great industry and acuteness in its author, and we expect much useful information from its perusal.

